# Supplementary material for: SH-29 and SK-119 Attenuates Air-Pollution Induced Damage by Activating Nrf2 in HaCaT Cells
Source: Int J Environ Res Public Health. 2021 Nov 24;18(23):12371. doi: 10.3390/ijerph182312371 (PMC8656889; doi:10.3390/ijerph182312371)
Supplement: Supplementary file 1 [file ijerph-18-12371-s001.zip › ijerph-1438464-supplementary.pdf]

SH-29 and SK-119 Attenuates Air-Pollution Induced Damage by Activating Nrf2 in HaCaT Cells

Shirin Kahremany [1,2], Lukas Hofmann [1], Noy Eretz-Kdosha [2], Eldad Silberstein [3], Arie Gruzman [1,*] and Guy Cohen [2,4,*]

[1] Department of Chemistry, Faculty of Exact Sciences, Bar-Ilan University, Ramat-Gan 5290002, Israel; shirin.kahremany@live.biu.ac.il (S.K.); lukas.hofmman@biu.ac.il (L.H.)
[2] The Dead Sea and Arava Science Center, The Skin Research Institute, Masada 8691000, Israel; noy@adssc.org
[3] Department of Plastic Surgery, Soroka University Medical Center, Ben-Gurion University of the Negev, Beer-Sheva 8410100, Israel; eldads@bgu.ac.il
[4] Eilat Campus, Ben Gurion University of the Negev, Eilat 8855630, Israel
* Correspondence: gruzmaa@biu.ac.il (A.G.); guy@adssc.org (G.C.)

This PDF file includes:

I. Scheme S1: Synthesis of SH-29 and SK-119 2
II. NMR spectra 3
III. Mass Spectrometry analytical data 7

I. Scheme S1: Synthesis of SH-29 and SK-119

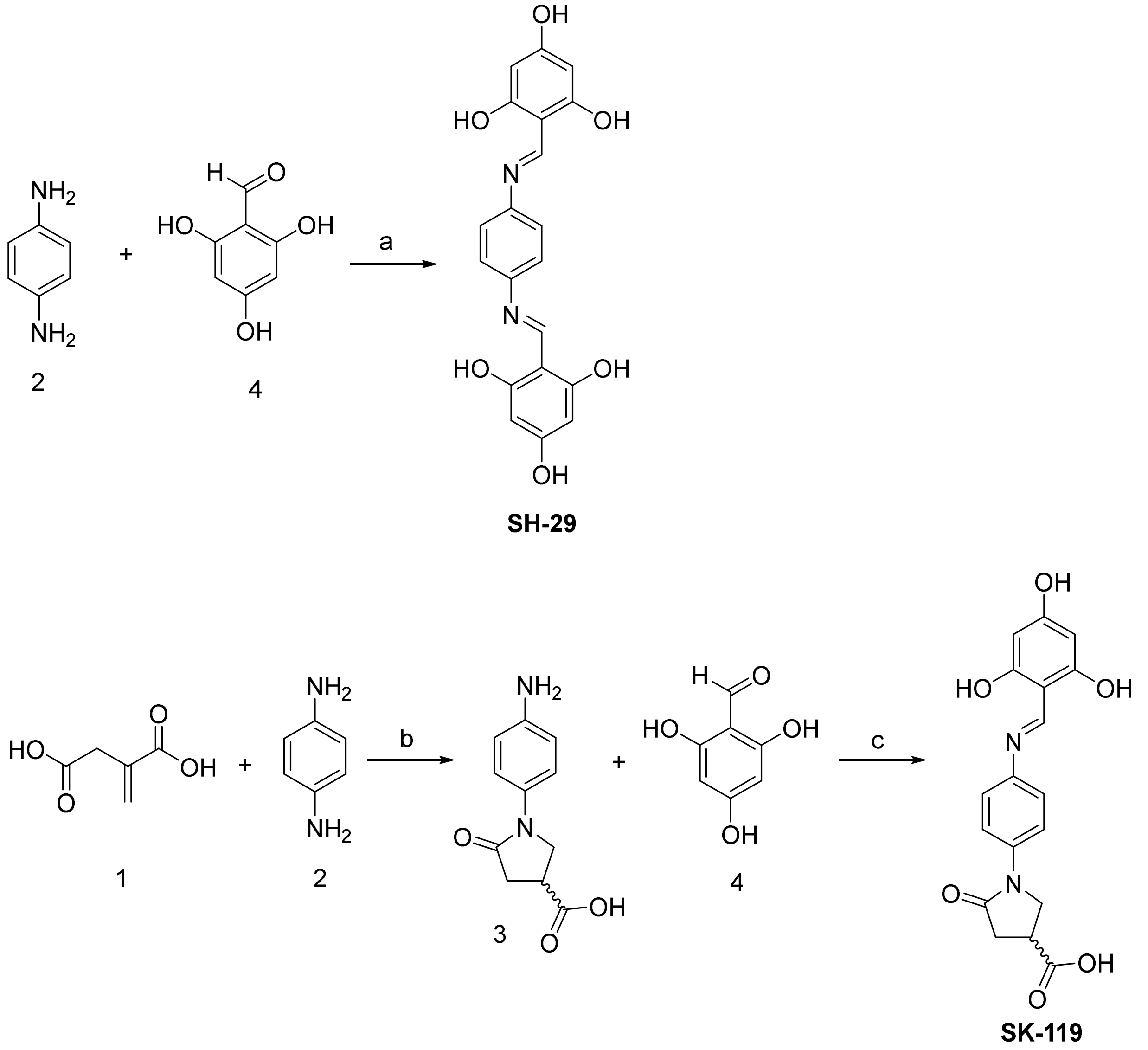

Synthetic procedure for the preparation of compounds SH-29 and SK-119. a) phenylenediamine (2), 2,4,6-trihydroxybenzaldehyde (4), EtOH, 5 h, r.t. b) itaconic acid (1), phenylenediamine (2), H_2O, reflux 1 h. c) EtOH, compound 3, 2,4,6-trihydroxybenzaldehyde (4), RT overnight.

II. NMR spectra

2,2'-((1E,1'E)-(1,4-phenylenebis(azaneylylidene))bis(methaneylylidene))bis(benzene-1,3,5-triol), **SH-29**

I. ^{1}H-NMR (400 MHz, DMSO)

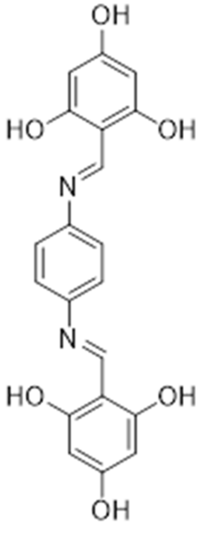

SH-29

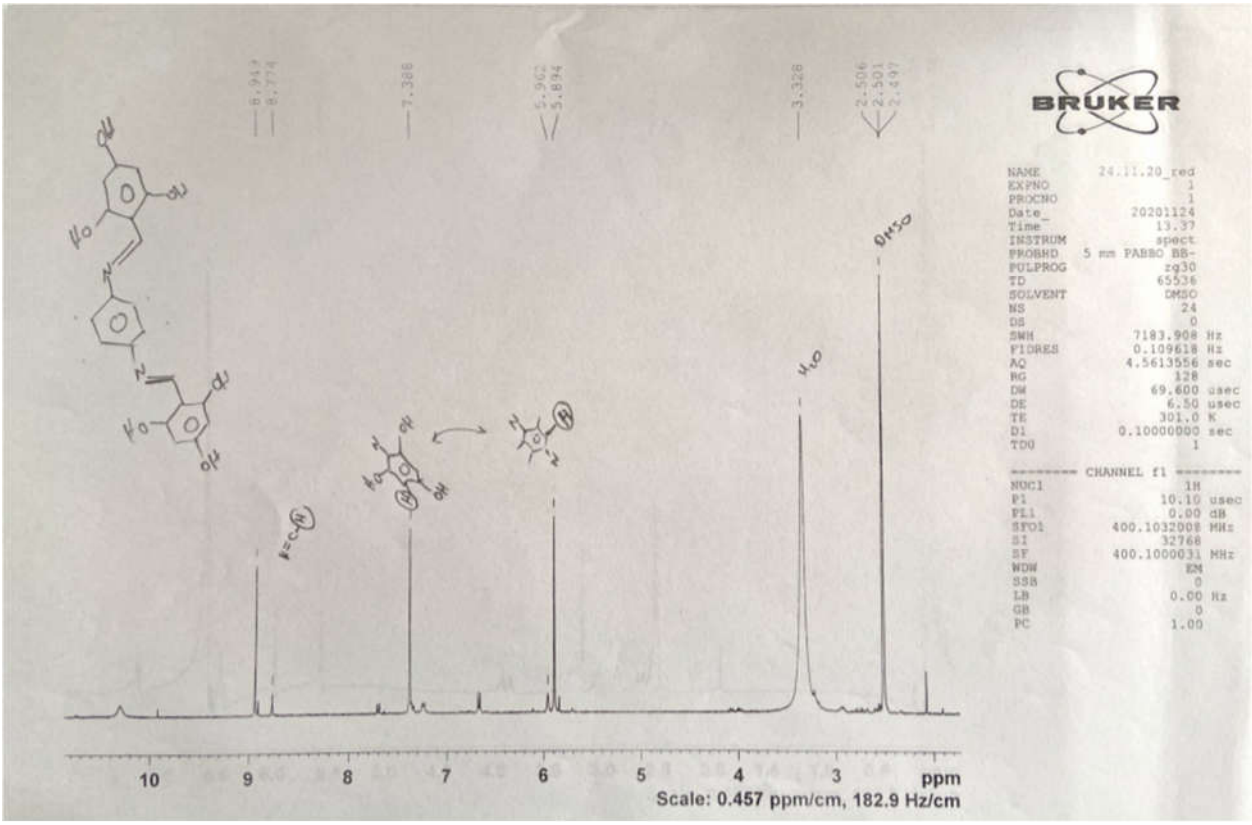

II. ^{13}C-NMR(400 MHz, DMSO)

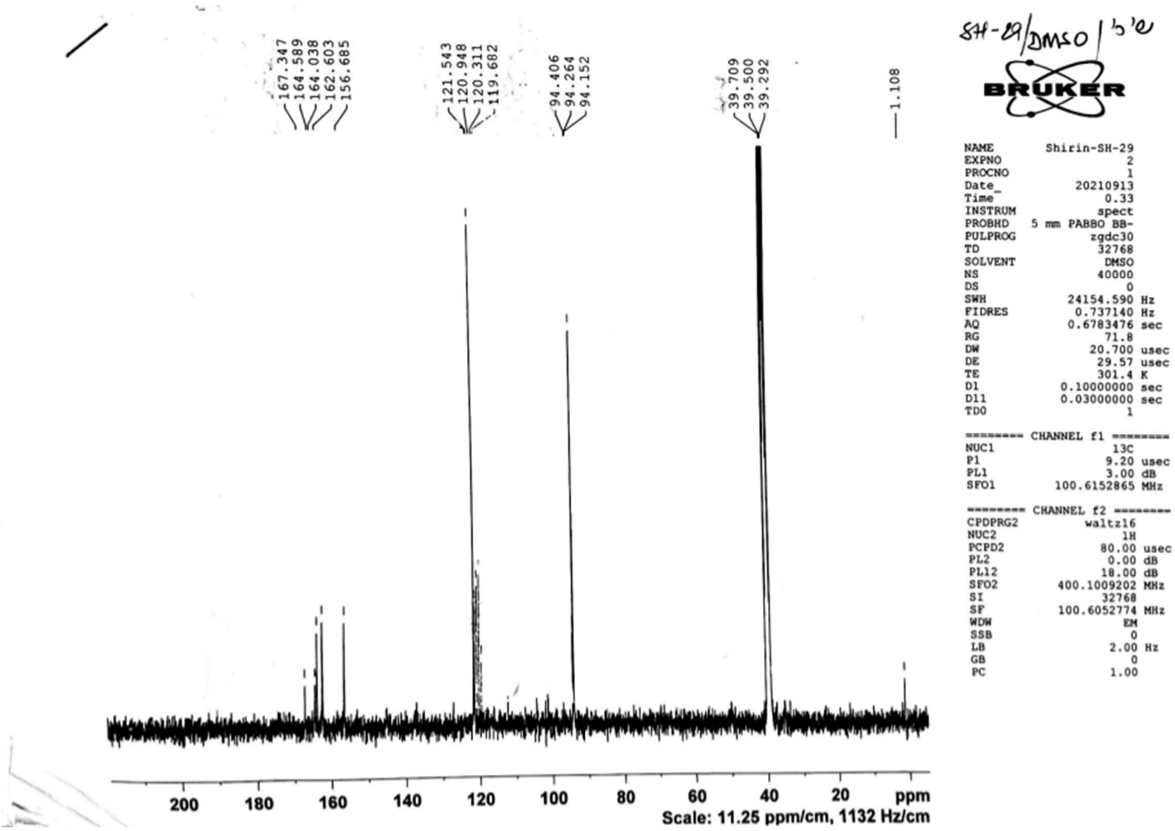

NMR assignment was previously reported [1]

5-oxo-1-(4-((2,4,6-trihydroxybenzylidene)amino)phenyl)pyrrolidine-3-carboxylic acid, **SK-119**

III. ^{1}H-NMR (400 MHz, DMSO)

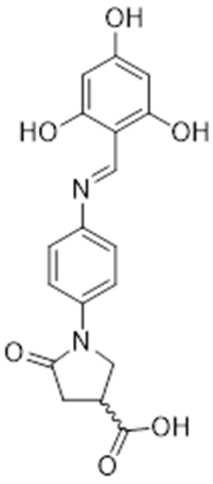

SK-119

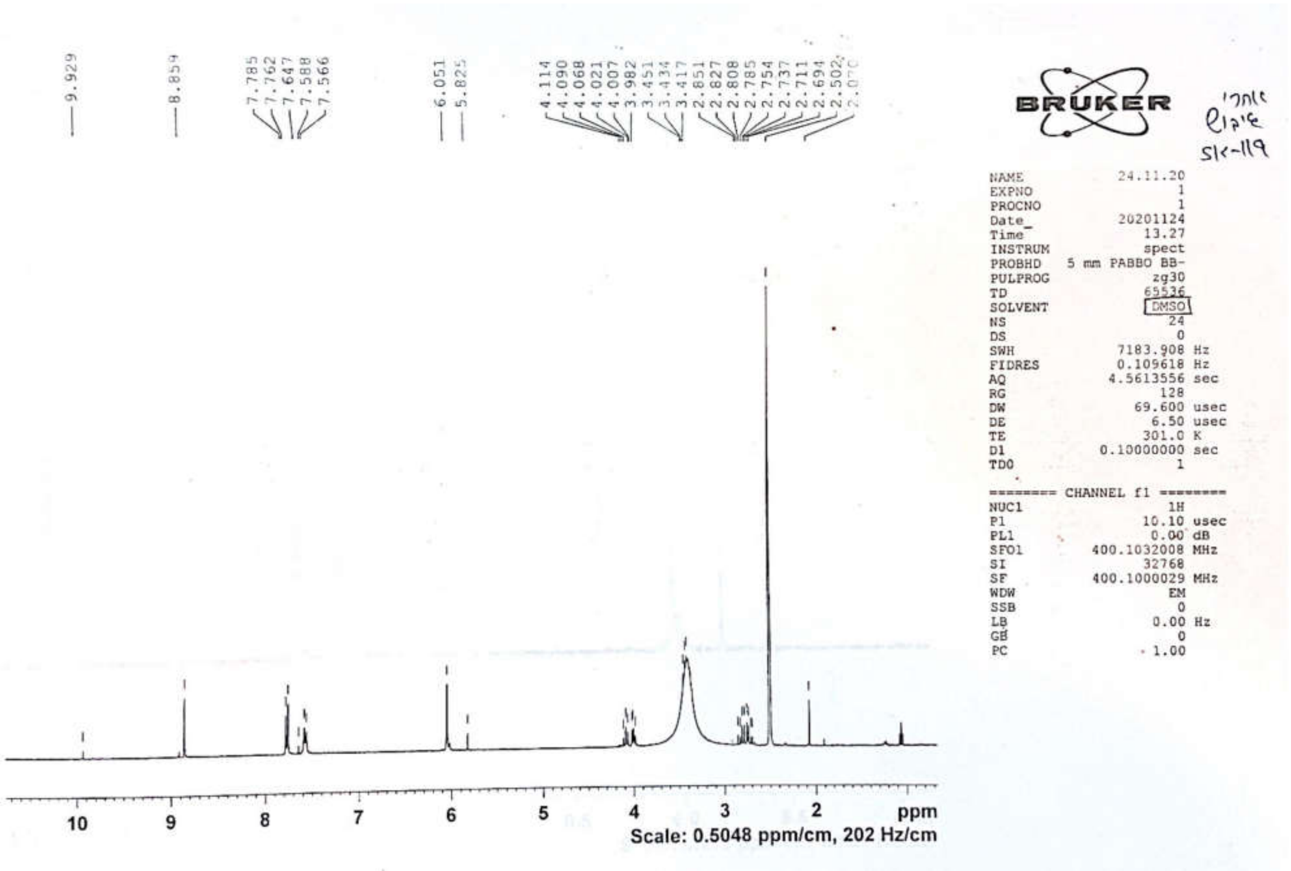

IV. ^{13}C-NMR(400 MHz, DMSO)

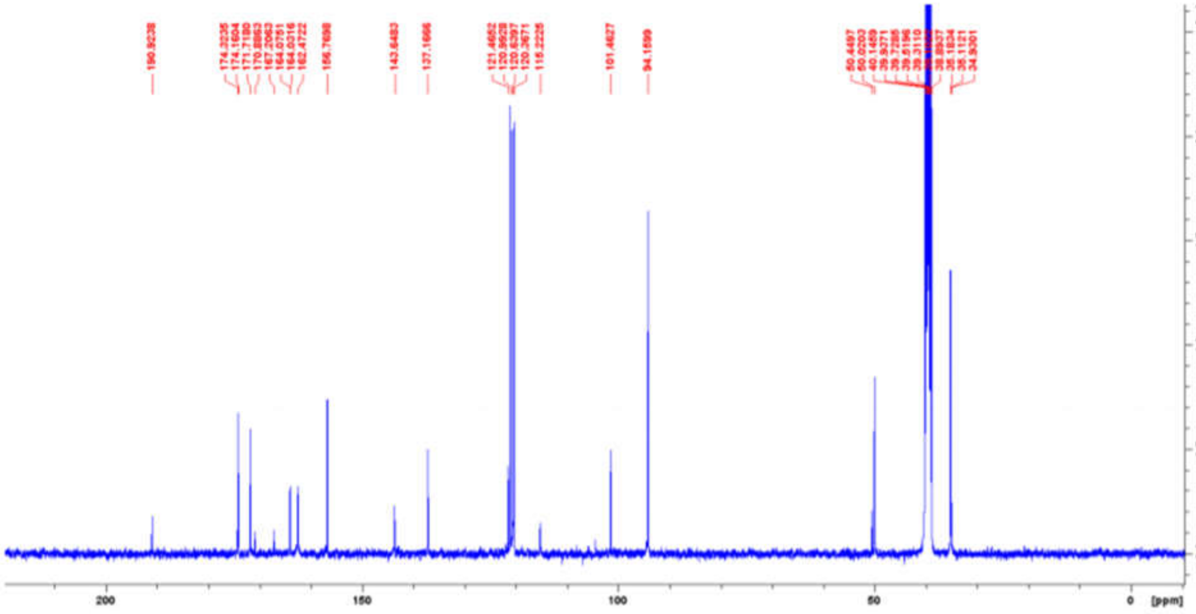

III. Mass Spectrometry analytical data

2,2'-((1E,1'E)-(1,4-phenylenebis(azaneylylidene))bis(methaneylylidene))bis(benzene-1,3,5-triol), **SH-29**

Qualitative Compound Report

Compound Label	m/z	RT	Algorithm	Mass
Cpd 1: C20 H16 N2 O6	379.093	0.6	Find By Formula	380.1007

MS Spectrum

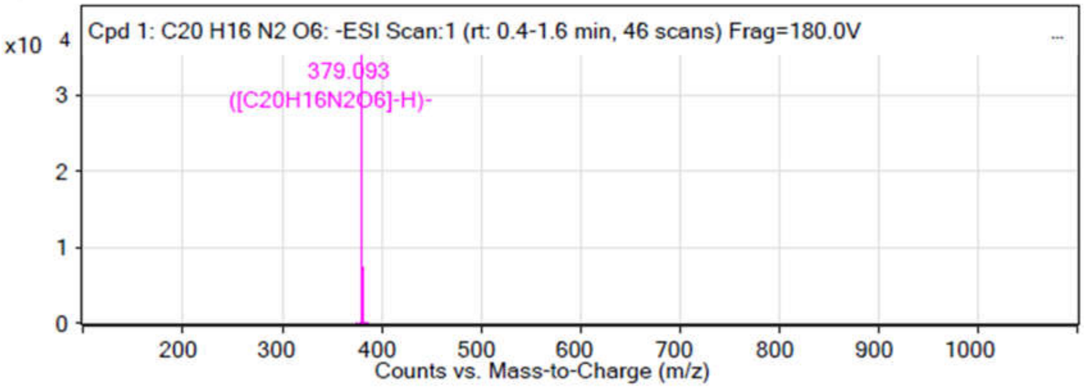

MS Zoomed Spectrum

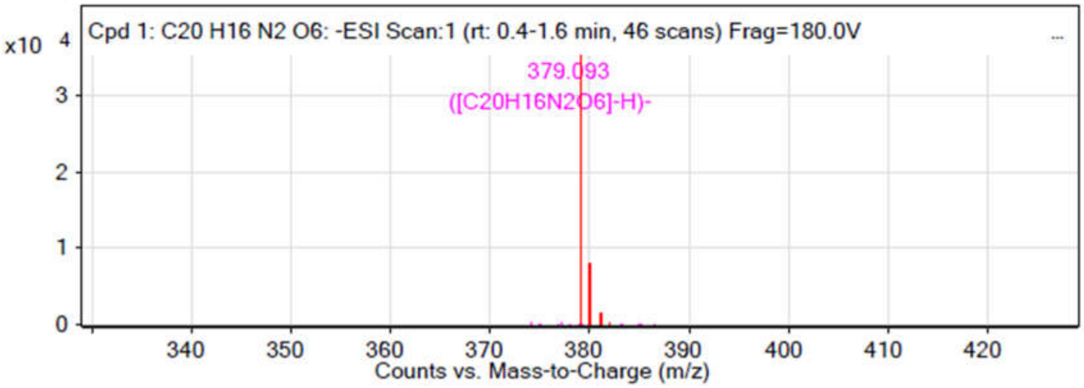

MS Spectrum Peak List

m/z	Calc m/z	Diff(ppm)	z	Abund	Formula	Ion
379.093	379.094	0.16	1	35212.06	C20H16N2O6	(M-H)-
380.096	380.097	0.77	1	7288.87	C20H16N2O6	(M-H)-
381.099	381.099	1.22	1	1100.89	C20H16N2O6	(M-H)-
382.102	382.102	-0.91	1	113.14	C20H16N2O6	(M-H)-

--- End Of Report ---

Qualitative Analysis Report

User Spectra

Spectrum Source	Fragmentor Voltage	Collision Energy	Ionization Mode
Peak (1) in "- TIC Scan"	250	0	ESI

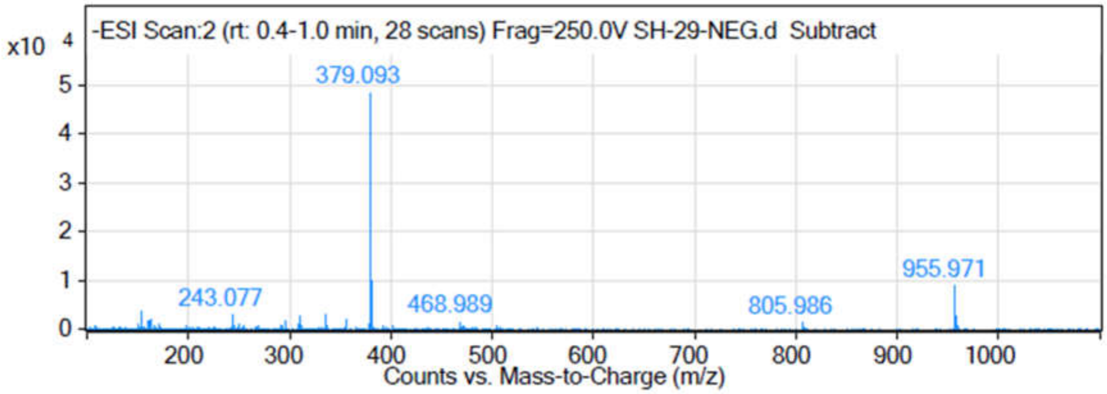

Peak List

m/z	z	Abund
92.928		1505.03
94.925		1277.5
108.045		726.11
151.004		788.85
153.019		3569.66
159.056		1416.09
160.842		1710.21
162.839		1740.31
164.836		667.46
170.944		868.28
199.087		759.8
243.077	1	3133.6
251.082		1016.61
255.077		764.34
295.072		1648.5
309.088		915.39
311.103	1	2854.08
335.103	1	3125.65
336.107	1	727.27
337.084		694.88
355.093		1781.02
377.075		1075.16
379.093	1	48275.35
380.096	1	9793.72
381.099	1	1617.79
468.989		1390.2
805.986		1208.88
955.971		9066.02
956.973		1638
957.968		2933.25

Compounds

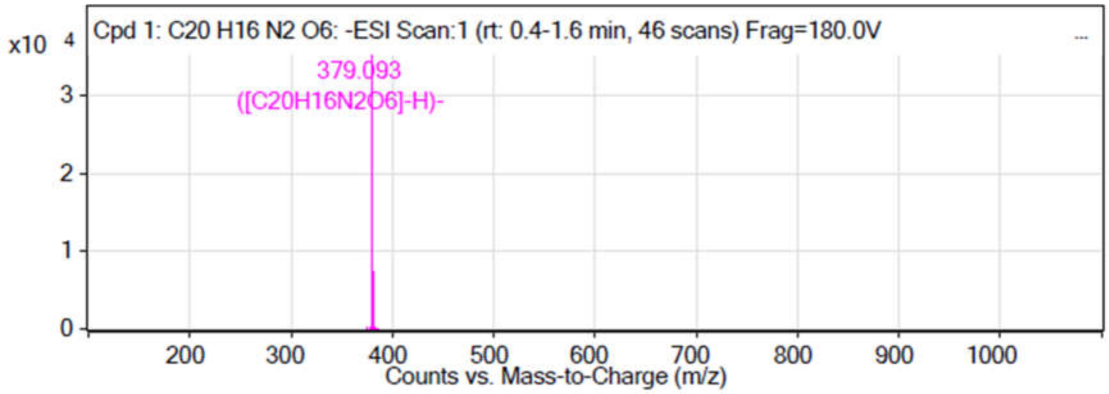

Peak List

m/z	z	Abund	Formula	Ion
379.093	1	35212.06	C20H16N2O6	(M-H)-
380.096	1	7288.87	C20H16N2O6	(M-H)-
381.099	1	1100.89	C20H16N2O6	(M-H)-
382.102	1	113.14	C20H16N2O6	(M-H)-

--- End Of Report ---

5-oxo-1-(4-((2,4,6-trihydroxybenzylidene)amino)phenyl)pyrrolidine-3-carboxylic acid, **SK-119**

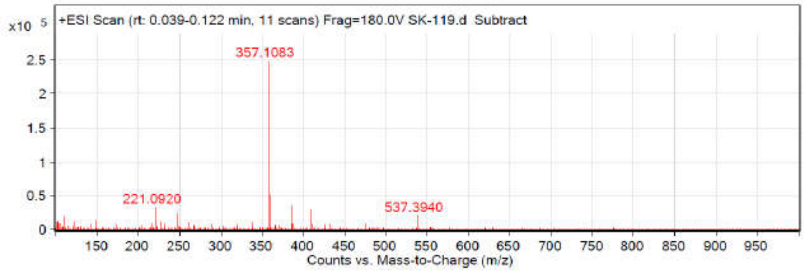

Peak List

m/z	z	Abund
54.9468		4160.51
55.9344		6869.46
56.9419		64186.87
72.937		12550.21
81.9371		48367.74
83.9404		5330.04
84.9463		5975.98
85.9415		6384.69
87.0441		14745.55
89.0595		3620.91
95.9525		5388.94
95.9731		4318.41
97.9684		72220.02
98.9614		27334.29
99.9687		9020.65
100.9566		11021.09
102.1276		12685.8
103.9555		8771.66
105.0697		9720.54
108.0673		3966.32
109.9432		19460.7
114.0913		4514.68
121.0741		3867.01
122.9639		12534.44
126.9672		3780.38
130.1588		4546.47
141.9585		7713.45
149.023		13540.89
172.8616		7416.14
202.8722		6104.05
217.1054		8517.03
221.092	1	32481.44
222.096	1	4056.02
227.1256		11458.43
230.8896		8376.76
246.8622		23633.44
248.8626	4	4188.94
261.1093		10435.04
261.1282		6657.95
267.1219		5732.12
288.2892		7180.11
301.1414		4964.74
318.7921		7068.65
337.1051		10102.43
357.1083	1	248457.59
358.1112	1	51375.8
359.1137	1	7545.01
365.1356		6329.38
371.0997		5591.6
385.1391		6876.68
387.1803	1	35066.03
388.1838	1	8342.91
409.1619	1	29193.48
410.1653	1	6899.88
425.1358		7783.54
432.2377	1	7886.26
475.113	1	9510.22
537.394	1	20491.01
538.3974	1	6725.91
553.3789		3628.97

Reference:

1. Kahremany, S.; Babaev, I.; Gvirtz, R.; Ogen-Stern, N.; Azoulay-Ginsburg, S.; Senderowitz, H.; Cohen, G.; Gruzman, A. Nrf2 Activation by SK-119 Attenuates Oxidative Stress, UVB, and LPS-Induced Damage. *Skin Pharmacol Physiol* **2019**, *32*, 173-181, doi:10.1159/000499432.
